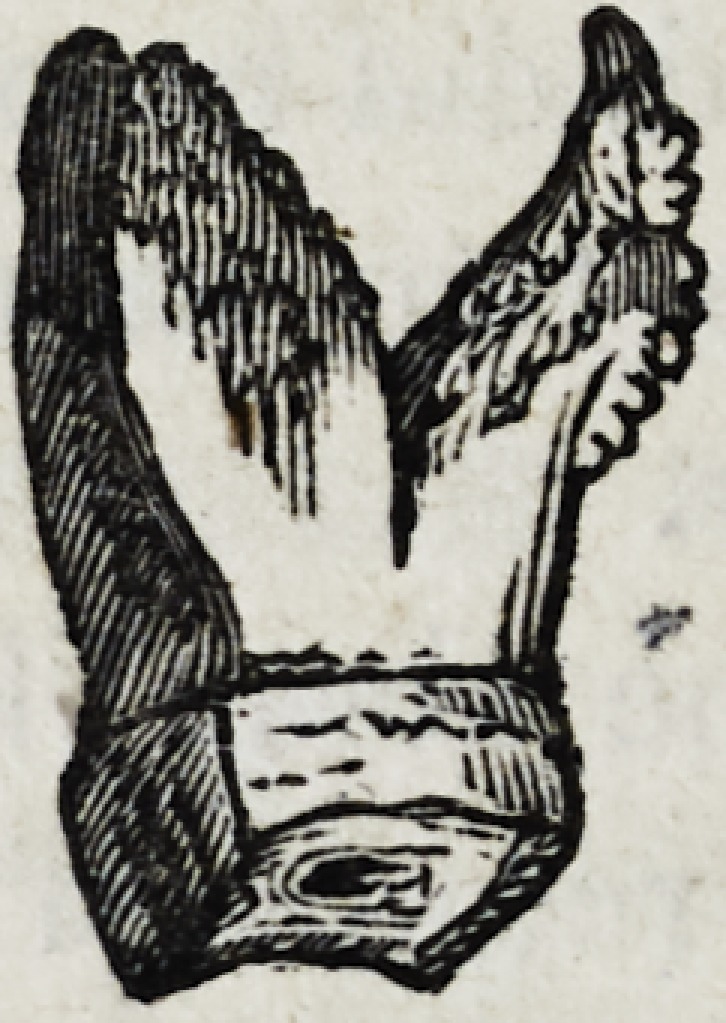# Case of Ozæna, Accompanied by Frequent Paroxysms of Neuralgia Faciei, Cured by the Extraction of a Tooth

**Published:** 1841-12

**Authors:** Chapin A. Harris

**Affiliations:** Baltimore.


### ARTICLE IV.

*Case of Ozæna, accompanied by frequent paroxysms of Neuralgia Faciei, cured by the Extraction of a Tooth.* By CHAPIN A. HARRIS, M. D. Baltimore.

MR. S——, a resident of a neighbouring county, of a full habit, and slightly disposed to scorbutus, had, for a little more than two years, been the subject of an obstinate and distressing affection of the left nasal fossa, and of frequent attacks of pain, which he represented as being, at times, almost excruciating—commencing immediately over the first left superior molaris, thence shooting back to the angle of the jaw, then to the ala of the nose, inner angle of the eye, and not unfrequently to the top of the head. Ulceration had taken place in the mucous membrane of the affected nostril, and a thin fetid matter, occasionally streaked with pus and blood, was almost constantly discharged, excoriating the parts with which it came in contact. The cavity of the nostril had become so much closed by the thickening of its membranes, that the passage of air through it was prevented ; the external integuments had assumed a dark florid appearance, and become considerably tumefied and sensitive to the touch.

His teeth having been suspected, though to all appearance perfectly sound, as having some agency in the production of the neuralgic affection, he was directed to a dentist to have them examined, but as none of them exhibited any signs of decay, it was thought to be dependent upon some other cause. Accordingly the remedial means usually employed for this, as well as those for the other affection under which he was labouring, were prescribed; but from their use, although continued for several months, and under a variety of modifications, he derived no benefit.

His complaints becoming more and more aggravated, he at length became apprehensive as to their result, and determined by the advice of his friends, to visit several of the medicinal springs in Virginia. At one of these, he met with an eminent medical gentleman from one of the northern cities, whom he consulted, but neither from his prescription nor the use of the waters of any of the springs that he visited, did he obtain the slightest relief, and after remaining from home two months, he returned in a state almost bordering on despair.

To add to his affliction, he about this time, began to be annoyed with a constant pain in the region of the antrum of the affected side. This, in connection with a soreness in a tooth immediately beneath, which he had felt throughout the whole course of his protracted and complicated disease, but which had not until now been sufficiently great to attract particular observation, soon led to the discovery of the cause both of the nasal and neuralgic affections, and also to the means by which they were finally cured. The pain in his jaw continuing to increase, and from its resemblance to tooth-ache, he was induced, September 9th, 1839, to apply to me for advice. From the description which he gave of it and the other circumstances connected with the case, the belief that the antrum was diseased, and that a morbid condition of some one or more of his teeth or their sockets, had been chiefly instrumental in its production, at once forced itself upon me. With a view of satisfying myself more fully on this point, I gave his mouth a careful examination. His teeth, at least so far as their crowns were concerned, were all free from disease, but the socket of the first left superior molaris, which was that of the sensitive tooth, was considerably wasted—the tooth itself, particularly its outer and posterior surfaces, thickly coated with tartar, slightly loosened and partially protruded from the jaw; whilst the surrounding gum was inflamed and spongy. The tooth having thus, as it would seem, from some causes or other, become obnoxious to the parts within which it was contained, and as it had no antagonist, its removal appeared to constitute the first and principal indication of cure. To this, upon its being advised, he readily submitted. The operation was followed by a sudden gush of thin fetid matter from the antrum, which communicated with the socket of the tooth by an opening sufficiently large to admit of the easy introduction of the end of a small goose-quill, and a subsidence of pain. The cause of his complicated malady was now revealed. The roots of the tooth, as may be seen from the following drawing, which is an exact representation of the tooth, were found to be greatly enlarged by exostosis.

The intervening transverse and longitudinal alveolar walls had been destroyed, and the place which they had formerly occupied filled with fungus. The edges of the surrounding wall were considerably wasted, and its surface interiorly, rough and enlarged.

A strong solution of *argentum nitratum* having been applied to the diseased socket, by means of a camel's hair pencil, and the antrum syringed out with diluted tinct. myrrh, which last was directed to be repeated twice a day as long as the opening into that cavity should remain unclosed, the balance of the cure was entrusted to the restorative energies of the economy.

The following day he left the city, and I heard no more of him for six weeks; at the expiration of which time he again visited it, and called to inform me of the amendment that had taken place in his condition. He was now able to breathe through his left nostril almost as freely as the right—the discharge from it was greatly diminished and of a more healthy character. He had had but one return of his neuralgic affection, which occurred the fourth day after the removal of the tooth, and was less severe than any of the former paroxysms. The opening into the antrum had closed, and the socket was rapidly filling with healthy granulations.

December 3d. I again had the satisfaction of seeing him and of being informed that every vestige of his nasal and neuralgic affections had disappeared.

REMARKS.

The circumstances connected with the history of the foregoing case would seem to justify the conclusion, that the irritation produced by the enlargement of the roots of the tooth, had given rise to a morbid excitement in the mucous membrane of the antrum maxillary—that this had extended to that of the left nostril, where the parts being more exposed to external irritating agents, had taken on a new and more aggravated form of disease; and that the neuralgia was the result of the irritation in the nose, antrum or socket, and most probably the last. How far the deposition of tartar that had formed on the tooth may have been accessory to the exostosis, is a question perhaps not easily solved. That it might produce such an effect can very readily be conceived, for when we take into consideration the morbid influence the presence of this substance frequently exerts upon the secretions of the mouth, the gums and alveolar processes, it will not appear at all strange that it should give rise to this. The disease being dependent on inflammation of the periostia of the roots of the teeth, may be brought on, when favoured by a constitutional tendency, by any thing producing preternatural excitement in these membranes, and that salivary calculus often does this, is a fully recognized axiom in dental pathology. But how far it may have been concerned, either primarily or secondarily, in its production in this instance, I will not take upon myself to determine, inasmuch as there was one other circumstance connected with the history of the case, that may have been the primary cause of the whole disturbance. That was, the want of an opposing tooth against which for this to act; and it may be well here to remark, that whenever this happens, especially to a superior molaris, and in the present case, it had existed, as I was informed, for about seven years, the surrounding gum is apt to become inflamed, the periosteum of its roots morbidly excited, and the socket to waste and sometimes to become gradually filled with ossific depositions,* as though nature,

* The doctrine that teeth, after having lost their antagonists, are sometimes partially displaced by the gradual filling up of their sockets at the bottom, conscious that the organ was of no further use, exerted her energies to expel it from the jaw. This tendency, every dentist of observation and experience must have noticed, and Dr. Koecker, a distinguished European practitioner, in accordance with what would thus seem to be a law of the economy, recommends the extraction of all such teeth; but, as there are frequent instances where, by proper attention to their cleanliness, they may be permitted to remain with impunity, this advice should not always be followed.—*Md. Med. and Surg. Jour.*

## Figures and Tables

**Figure f1:**